# Predictive modelling for COVID-19 outbreak control: lessons from the navy cluster in Sri Lanka

**DOI:** 10.1186/s40779-021-00325-4

**Published:** 2021-05-18

**Authors:** N. W. A. N. Y. Wijesekara, Nayomi Herath, K. A. L. C. Kodituwakku, H. D. B. Herath, Samitha Ginige, Thilanga Ruwanpathirana, Manjula Kariyawasam, Sudath Samaraweera, Anuruddha Herath, Senarupa Jayawardena, Deepa Gamge

**Affiliations:** 1 Disaster Preparedness and Response Division, Ministry of Health, 385, Rev. Baddegama Wimalawansa Thero Mawatha, Colombo, 01000 Sri Lanka; 2 Epidemiology Unit of the Ministry of Health, Colombo, 01000 Sri Lanka; 3 Sri Lanka Navy, Colombo, 01100 Sri Lanka

**Keywords:** COVID-19, Predictive modelling, SIR model, Navy cluster, Outbreak management

## Abstract

In response to an outbreak of coronavirus disease 2019 (COVID-19) within a cluster of Navy personnel in Sri Lanka commencing from 22nd April 2020, an aggressive outbreak management program was launched by the Epidemiology Unit of the Ministry of Health. To predict the possible number of cases within the susceptible population under four social distancing scenarios, the COVID-19 Hospital Impact Model for Epidemics (CHIME) was used. With increasing social distancing, the epidemiological curve flattened, and its peak shifted to the right. The observed or actually reported number of cases was above the projected number of cases at the onset; however, subsequently, it fell below all predicted trends. Predictive modelling is a useful tool for the control of outbreaks such as COVID-19 in a closed community.

Infectious diseases in semi-confined places such as Cruise Ships, Military Barracks, and College Dormitories can have rapid spread, coronavirus disease 2019 (COVID-19) being no exception [1–3].

While security forces are essential to staying in confined places as per their operational requirements, the spread of COVID-19 could have serious repercussions not only on the staff of the forces, but also on national security. On 22nd of April 2020, a new case from Polonnaruwa district, a navy sailor attached to the Sri Lanka Navy Base at Welisara, on leave tested positive for COVID-19 [4]. Subsequently increasing number of COVID-19 cases were diagnosed from the same Naval Base. Having confirmed an outbreak in the Naval Base, several public health measures were implemented by the Epidemiology Unit of the Ministry of Health together with Sri Lanka Navy based on the findings of epidemiological investigations. It was necessary to predict how the outbreak could spread among the confined space of the Navy Base, in order to understand the health system demand from the infected individuals, as well as to quantify the effects on operational continuity of the Navy. The objective of this joint exercise by the civil and naval health authorities was to predict the possible number of cases within the susceptible population in Sri Lanka Navy Base at Welisara and their associated operational units in the Western Province, to be used primarily for operational planning purposes by the Ministry of Health to control a possible widespread outbreak of COVID-19 in Sri Lanka.

The COVID-19 Hospital Impact Model for Epidemics (CHIME) developed by Predictive Health Care Team at Penn Medicine, Philadelphia, USA, which uses a Monte Carlo simulation instantiation of a susceptible, infected, removed (SIR) model with a 1-day cycle was utilized [5]. The model was run on 20th May 2020 for a susceptible population of 10,400, with the number of hospitalized patients on the day of running the model being 357, the first case hospitalized on 22nd April 2020, and social distancing being implemented on 26th April 2020. Social distancing scenarios of 0, 25, 50, and 74% were run with 10 days of infectious period and 30 days of the projection period.

As shown in Fig. 1, with an increasing social distancing from 0 to 74%, the epidemiological curve flattened, while the peak of the curve shifted right. Further, it could be seen that the observed or actually reported number of cases was well above the projected number of cases up to 7th May 2020. However, beyond this date, the reported number of cases was lower than the projected number of cases from the model under four social distancing scenarios considered. The typical bell-shaped epidemiological curve was not observed among the observed or the actual COVID-19 cases. This could most likely be due to the vigorous interventions carried out to curtail the outbreak by the health and security sectors.

**Fig. 1:**
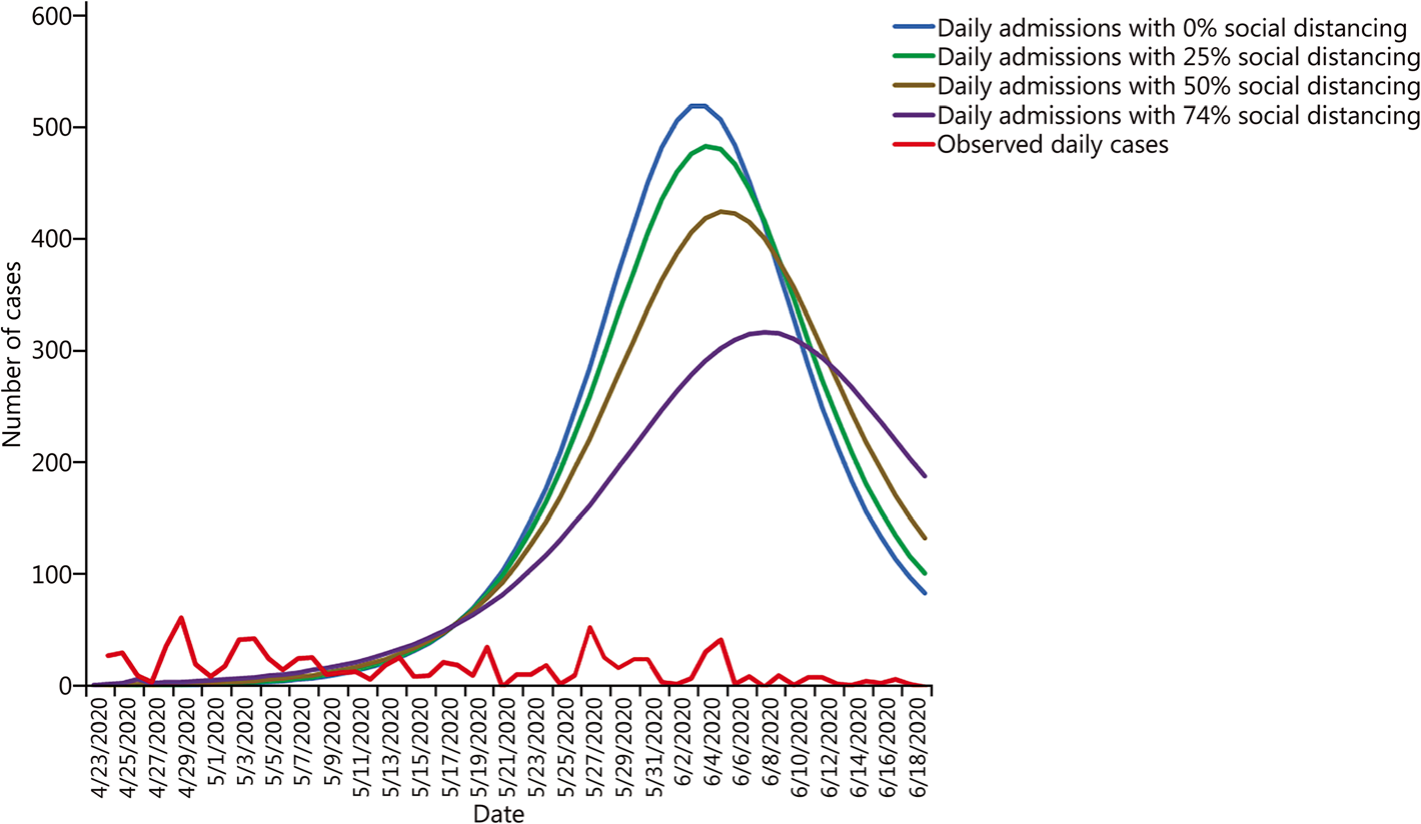
Projected and observed daily cases*.* The projected daily patients under 0, 25, 50 and 74% social distancing scenarios are shown in blue, green, brown and purple lines respectively. The projected daily patient curve flattens with increasing social distancing scenarios, while the time to the peak prolongs. The red line shows the observed daily cases which continue to be above the projected daily patient lines of all four social distancing scenarios up to 7th May 2020. However, since this date, the red line showing the observed number of cases curve goes below the projected number of cases under four social distancing scenarios considered

The practical use of a prediction model made readily available through an online open-source platform for the operational aspects of controlling outbreaks such as COVID-19 in a closed community was evident from this exercise. The findings could be used for strategic planning for curtailing the outbreak and for health system preparedness for the current outbreak, as well as for those in the future. Although active epidemiological surveillance, contact tracing, case isolation, and case management should be the cornerstone of outbreak management, the predictive epidemiological modelling could supplement the above efforts. It is recommended that both health and security personnel are exposed to the use of predictive epidemiological modelling during their outbreak preparedness and response training.

## Data Availability

The datasets used and/or analysed during the current study are available from the corresponding author on reasonable request.

## Abbreviations

COVID-19: coronavirus disease 2019
SIR: susceptible, infected and removed
CHIME: COVID-19 Hospital Impact Model for Epidemics
